# Chemotherapy Strategies and Their Efficacy for Mesenchymal Chondrosarcoma

**DOI:** 10.3390/curroncol32110615

**Published:** 2025-11-03

**Authors:** Piotr Remiszewski, Julia Wąż, Sławomir Falkowski, Piotr Rutkowski, Anna M. Czarnecka

**Affiliations:** 1 Department of Soft Tissue/Bone Sarcoma and Melanoma, Maria Sklodowska-Curie National Research Institute of Oncology, 02-781 Warsaw, Poland; 2 Medical Faculty, Medical University of Warsaw, 02-781 Warsaw, Poland; 3 Department of Experimental Pharmacology, Mossakowski Medical Research Institute, Polish Academy of Sciences, 02-781 Warsaw, Poland; 4 Outpatient Chemotherapy Department, Maria Sklodowska-Curie National Research Institute of Oncology, 02-781 Warsaw, Poland

**Keywords:** mesenchymal chondrosarcoma, perioperative chemotherapy, anthracyclines, HEY1-NCOA2 fusion, neoadjuvant treatment, adjuvant therapy

## Abstract

Mesenchymal chondrosarcoma (MCS) is characterised by small round cell biology, frequent *HEY1-NCOA2* fusion, and high vascularity. These features plausibly lessen extracellular matrix barriers and confer relative chemosensitivity. We synthesised peri-operative (preoperative/neoadjuvant; postoperative/adjuvant) and palliative chemotherapy outcomes separately across multiple cohorts and case reports as well as the summarised the guidelines (ESMO/NCCN) In localised disease, integrating multi-agent Ewing-type chemotherapy with complete resection is associated with improved disease control. Contemporary 5-year overall survival (OS) typically spans ~55–73% across studies, while event-free survival (EFS) gains are demonstrated more consistently than OS gains in pooled analyses. In advanced MCS, first-line polychemotherapy yields modest, non-curative activity, with objective response rates (ORRs) of ~25–35% in adults, median progression-free survival (PFS) of ~4.7–6.7 months, and median OS of ~18 months. Activity may be higher in younger patients and for platinum–anthracycline combinations. We also discussed emerging therapies. Trabectedin demonstrates low disease control rate in translocation-related sarcomas, including few MCS cases. Anti-angiogenic tyrosine kinase inhibitors, such as apatinib and pazopanib, demonstrate activity in chondrosarcoma, but MCS-specific data are lacking. *IDH1* inhibition benefits conventional subtypes rather than MCS. Early immunotherapy experience is limited, but pathway-directed strategies targeting *BCL2* and *PI3K-mTOR* warrant evaluation.

## Simple Summary

Mesenchymal chondrosarcoma (MCS) is characterised by small round cell biology with frequent *HEY1-NCOA2* fusion, early haematogenous spread, and greater sensitivity to systemic chemotherapy (CHT) than other chondrosarcoma subtypes. CHT is used in both the peri-operative (neoadjuvant and adjuvant) and metastatic settings. Recommended regimens are similar to those used in Ewing’s sarcoma and include vincristine, doxorubicin, and cyclophosphamide (VDC, sometimes abbreviated as VAdriaC/IE), ifosfamide and etoposide (IE). Gemcitabine–docetaxel (GD) is a later-line option in metastatic settings. We thoroughly reviewed retrospective and prospective studies (primarily on novel immunotherapies and targeted therapies) and case reports to evaluate treatment outcomes, including overall survival (OS) and progression-free survival (PFS), as well as the CHT regimens used. The strategies used in neoadjuvant, adjuvant and palliative CHT (first and subsequent lines) were discussed separately.

## 1. Introduction

**Figure 1:**
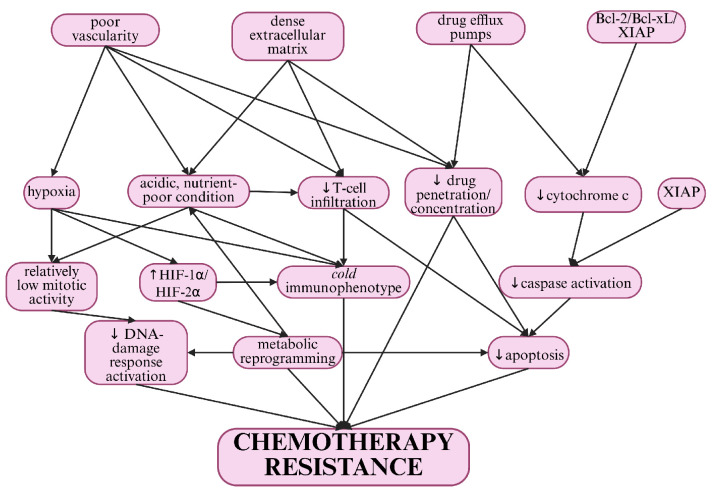
Cell-intrinsic and microenvironmental pathways underpinning chemoresistance in chondrosarcoma. This is a conceptual map of drug-tolerance mechanisms in chondrosarcoma. It highlights the factors that impede drug penetration, such as low proliferative fractions, a dense cartilage matrix and hypoxia. It also shows signalling pathways, such as Notch signalling, as well as DNA-damage repair, drug-efflux pumps and putative stem-like niches. These factors collectively suppress cytotoxic efficacy. This schematic aims to explain why responses are modest compared with those of other small round cell tumours and will be referenced in later therapy tables. (Created with BioRender. Remiszewski P. et al. (2025), https://BioRender.com/fx2wht8, accessed on 11 July 2025.)

Chondrosarcoma (CS) is the most common primary malignant bone tumour in adults and is characterised by cartilaginous matrix production [1]. Mesenchymal chondrosarcoma (MCS) and dedifferentiated chondrosarcoma (DDCS) are characterised by aggressive clinical behaviour and poor prognosis [2]. These high-grade variants, representing a small proportion of CS, present unique challenges due to their high metastatic potential and limited response to standard therapies [3]. MCS, characterised by small round cells interspersed with islands of cartilaginous differentiation, is a rare and aggressive sarcoma affecting younger populations [4]. Despite its histologic similarity to other small round cell tumours such as Ewing’s sarcoma (ES), MCS has distinct biological and molecular features that complicate its management (Figure 1).

**Figure 2:**
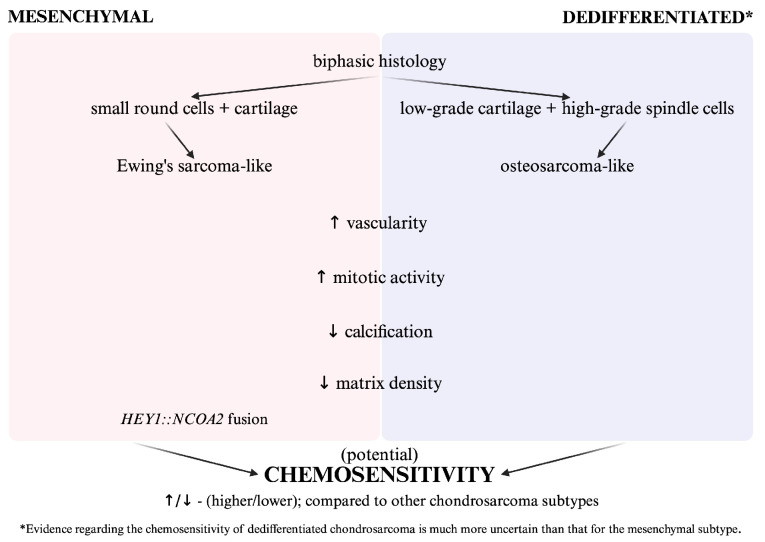
Tumour-intrinsic and microenvironmental factors shaping chemosensitivity in mesenchymal and dedifferentiated chondrosarcoma. Both mesenchymal chondrosarcoma (MCS) and dedifferentiated chondrosarcoma (DDCS) are biphasic. MCS comprises small, round areas of cartilage and typically harbours the HEY1::NCOA2 fusion gene. DDCS shows low-grade cartilage abutting a high-grade, non-cartilaginous sarcoma that is often osteosarcoma-like. Compared to other subtypes of chondrosarcoma, both display increased vascularity and mitotic activity, as well as decreased calcification and matrix density. Increased vascularity improves perfusion and drug delivery, while reduced calcification and matrix density lessen the extracellular matrix diffusion barrier. Higher proliferation also increases susceptibility to cell-cycle-dependent cytotoxics. Together, these features may explain the relatively higher, yet still modest, chemosensitivity, with stronger evidence in MCS and more uncertainty in DDCS. (Created with BioRender. Czarnecka, A. (2025) https://BioRender.com/cgzx7vw, accessed on 11 August 2025).

MCS is characterised by a small round-cell component driven by translocations and a richly vascular, haemangiopericytoma-like stroma [5,6,7,8,9]. Cartilage is relatively scarce and often poorly calcified. Together, these features lessen the extracellular matrix (ECM) diffusion barrier and hypoxia that impede drug access in conventional chondrosarcoma [5,6,7]. Meanwhile, higher cell turnover in the small-cell compartment increases vulnerability to cell-cycle-active agents. In practice, this biology aligns with the use of Ewing-type combinations (e.g., VDC/IE or VIDE). Overall, improved perfusion plus reduced matrix shielding—rather than a validated predictive biomarker—best explains the relatively greater, yet still modest, chemosensitivity of MCS (Figure 2). Studies often do not achieve statistical significance regarding the benefit of using chemotherapy (CHT) in mesenchymal or dedifferentiated chondrosarcoma (Graphical Abstract). Across retrospective series, evidence for chemotherapy (CHT) in MCS is mixed and often statistically non-significant, particularly when peri-operative and palliative settings are pooled. Contemporary multicentre data indicate that surgery with negative margins is the principal determinant of outcome: in an Australian cohort, 5-year overall survival (OS) was ~73% for localised cases, median OS ~104 months, while metastatic presentation showed far shorter survival (median ~35 months) [10]. Paediatric/AYA (adolescent and young adults) data suggest high resectability with good short-term survival (5-year OS ~89%; disease-free survival ~68%), yet responses to neoadjuvant CHT are typically limited to stable disease, underscoring relative chemoresistance also in these patients [11]. Registry-level analyses over long follow-up report favourable OS in localised disease after complete resection, with signals that anthracycline/alkylator-based CHT reduces local recurrence and associates with improved survival, although effect sizes vary and confounding is substantial [12]. Overall, the literature supports a surgery-centred approach in localised MCS (Table 1), while the incremental contribution of CHT remains context-dependent and uncertain; in metastatic disease, survival is markedly shorter and multimodality therapy may benefit selected patients [10,12]. Due to the fact that MCS-specific outcomes are often not reported and the literature limited, especially with the setting included (peri-operative/palliative), our aim is to provide a comprehensive narrative review of MSC treatment strategies that will be useful for clinical decision-making. We searched MEDLINE, Embase, Web of Science, Scopus, and ClinicalTrials.gov up until 31 August 2025. The strategy included terms: “dedifferentiated chondrosarcoma”, “chondrosarcoma”, “peri-operative”, “palliative”, “systemic therapy”, “chemotherapy”, “targeted therapy”, “survival”, and related synonyms, combined with Boolean operators (AND, OR, NOT) and truncation. Then, references from the relevant publications were used to identify further studies. We screened titles/abstracts and full texts to identify studies with MCS-specific outcomes reported in specific therapeutic contexts (neoadjuvant, adjuvant, first-line palliative, second- and further line(s) palliative). Data were extracted and narratively synthesised.

Surgical resection is the primary treatment for localised MCS, which has a more aggressive behaviour and poorer survival compared to conventional CS (Table 1) [13,14,15]. Surgery should aim for resection with disease-free margins; however, anatomical limitations may prevent wide margins, occasionally necessitating amputation. In such cases, NAC or AC may be considered, as non-radical resection carries a high risk of LR. Bishop et al. [16] aimed to characterize the clinicopathologic and radiographic features of MCS and evaluate the impact of various treatment modalities, particularly CHT, on patient outcomes.

**Table 1:** Key features and comparison of mesenchymal and conventional chondrosarcoma.

Chondrosarcoma Subtype	Mesenchymal	Conventional	References
Prevalence	1–10%	85–90%	[3,10,17,18,19]
Age of Onset	2nd–3rd decade	4th–6th decade	[10,11,18]
Histological Features	Small round cells with cartilaginous matrix and biphasic pattern	Lobulated cartilaginous matrix without dedifferentiation	[4,17]
Common Mutations	HEY1-NCOA2, PI3K/AKT, BCL2, SOX4, HES1, CDKN2A loss, TP53 alterations, NOTCH signalling alterations	IDH1, IDH2, COL2A1, TERT promoter mutations, RB1 loss, EXT1/EXT2 deletions	[3,19,20]
Metastatic Potential	High (lung, bone, soft tissue)	Low to moderate	[10,12,21]
Prognosis	Poor due to aggressive behaviour and metastasis risk (5-y OS of approximately 55%)	Moderate prognosis; better in localized disease (5-y OS of approximately 70%)	[10,18]

A side-by-side comparison of the prevalence, age at onset, histology, recurrent alterations, and outcomes of mesenchymal chondrosarcoma and conventional chondrosarcoma. Prevalence is approximately 1–10% versus 85–90%. Typical onset is in the second or third decade versus the fourth to sixth decade. Five-year overall survival is about 55% versus 70%. Abbreviations: BCL2: B-cell lymphoma 2; CDKN2A: cyclin-dependent kinase inhibitor 2A; COL2A1: collagen type II alpha-1 chain; EXT1/EXT2: exostosin glycosyltransferase 1/2; HEY1-NCOA2: HEY1-nuclear receptor co-activator 2 fusion; HES1: hairy and enhancer of split-1; IDH1/IDH2: isocitrate dehydrogenase 1/2; NOTCH: Notch signalling; OS: overall survival; PI3K/AKT: phosphoinositide 3-kinase/protein kinase B; RB1: RB transcriptional corepressor 1; SOX4: SRY-box transcription factor 4; TERT: telomerase reverse transcriptase; TP53: tumour protein p53.

## 2. Guidelines

The relationship between chemosensitivity and peri-operative use is a subject that merits further investigation. MCS is regarded as being chemo-sensitive. In the context of localised disease, the **ESMO** [19] recommends peri-operative chemotherapy (CHT)—that is, neoadjuvant chemotherapy (NAC) and/or adjuvant chemotherapy (AC)—using combinations of anthracyclines and alkylating agents (Table 2). This recommendation is classified as having an evidence level IV and a recommendation grade C. The recommended regimens typically consist of doxorubicin in combination with an alkylating agent, such as ifosfamide. However, ESMO does not specify a particular regimen. For widely metastatic high-grade chondrosarcoma, overall CHT benefit is limited; activity for gemcitabine–docetaxel has been reported in chondrosarcoma generally (IV, C). Specifically for MCS, trabectedin may be an option (V, C)—flagged as a low-level, selective consideration rather than standard of care. Targeted agents (e.g., pazopanib) and immunotherapy/IDH-pathway approaches are noted as investigational in chondrosarcoma; prospective trials are ongoing. In addition, ESMO recognises the use of RT for unresectable tumours, incomplete resections, or metastatic cases requiring local control [19].

**The NCCN** also does not provide MCS-specific cycle numbers in any setting; total cycles and dose intensity are individualised, following ES principles and patient tolerance [11]. In practice, centres commonly use vincristine-doxorubicin-cyclophosphamide (VDC) with ifosfamide-etoposide (IE)—an ES regimen. CHT is recommended as both NAC and AC to improve outcomes when combined with wide resection surgery. The NCCN does not endorse an MCS-specific alternative outside the ES framework; targeted agents cited for chondrosarcoma broadly are not MCS standards. Trial enrolment is encouraged. The application of metastasis-directed therapy should be selective, encompassing interventions such as pulmonary metastasectomy, stereotactic, or ablative RT. These should be administered within the context of an MDT, with a focus on balancing the burden, biology, and systemic control. ESMO and the NCCN engage in discourse on the local treatment of oligometastatic disease and the provision of symptom-directed palliation; decisions are made on an individualised basis [11].

While both guidelines emphasise the importance of systemic therapy alongside surgery, the NCCN [11] focuses more on standard protocols adapted from ES, while ESMO [19] offers a broader approach, allowing flexibility in CHT regimens and highlighting investigational therapies as additional options.

Importantly, extraskeletal myxoid CS (EMCS) represents a different entity and is classified as soft tissue sarcoma with a rather indolent course [22]. Therefore, it should not be managed with bone sarcoma guidelines and included into CS cohorts. If so, a clear distinction should be made to avoid confusion as differential diagnoses between MCS and EMCS are critical [22].

## 3. Neoadjuvant Chemotherapy

Unlike conventional CS, MCS is relatively chemosensitive; however, radiologic objective responses to neoadjuvant chemotherapy are uncommon and stable disease (SD) predominates. For example, the Cooperative German Studies (COSS/CWS) found objective tumour responses in 4 out of 7 young MCS patients treated preoperatively [16]. Italiano et al. observed an overall objective response rate (CR + PR) of 31% in MCS with first-line CHT (many receiving NAC), which was significantly higher than in conventional CS [12]. For MCS, robust histological necrosis rates after NAC are not well defined. Small paediatric/AYA (adolescent and young adults) series indicate that radiological response is uncommon and stable disease is typical, with rare partial responses; necrosis percentages were not systematically reported [21]. In this study for the small cell undifferentiated variant, patients received preoperative combination CHT and irradiation. For the hemangiopericytomatoid variant, preoperative CHT using T-10/T-11 protocols was given before definitive surgery. The T-10 consists of methotrexate, bleomycin, cyclophosphamide, dactinomycin (BCD), doxorubicin, cisplatin; T-11 uses same drug set (intensified sequencing). In five evaluable patients, this NAC approach showed encouraging results. The median OS was 37.9 months, and 28% of patients were alive at 10 years. No separate PFS, OS, or DFS figures were provided for the NAC group. One series reported that approximately 15% of MCS patients achieved ≥90% necrosis in the resected specimen after anthracycline-based NAC [23] which, while lower than seen in ES, indicates some chemosensitivity. Clinically, NAC can sometimes downsize the tumour to facilitate limb-sparing surgery or safer resection margins as commonly seen in specific subtypes soft tissue sarcomas [24].

It remains difficult to isolate the specific benefit of NAC in MCS from combined modality treatment. Evidence for a survival effect of peri-operative CHT (NAC ± AC) is retrospective. A systematic review of 107 patients reported longer event-free survival (EFS) with CHT (*p* = 0.046) and a non-significant OS trend (*p* = 0.139) [14]. Similarly, a European Musculoskeletal Oncology Society (EMSOS) study of 113 MCS patients reported a significantly lower risk of relapse and death in those treated with CHT (mostly multi-agent regimens) [17]. In this cohort, median PFS was 7 years and median OS was 20 years for patients receiving CHT (compared to much worse long-term outcomes historically without CHT). In the Japanese Musculoskeletal Oncology Group (JMOG) series (n = 57), 33% of localised cases received NAC and/or AC; among evaluable NAC recipients, partial response occurred in 1/4 and SD in 3/4. Five-year OS for all patients was 66% (localised 79%); NAC/AC was associated with a non-significant OS trend (90.9% vs. 72.6%, *p* = 0.107) but no MFS (metastasis-free survival) or LRFS advantage [25]. In contrast, in the series of 13 cases of head and neck MCS, treatment and outcome data were available for a subset of patients [26]. All patients underwent surgical resection; use of CHT was variable. Two patients received neoadjuvant chemotherapy; adjuvant/other systemic therapy was used selectively. One patient received an VDC/IE regimen in the NAC setting and showed no significant treatment effect in the resected specimen. The other patient received high-dose CTX, DOX, and VCR as NAC with no detectable response; this patient was subsequently treated with ETO and IFO postoperatively. Outcome data were available for seven patients with a median follow-up of 60 months (range, 12 to 193 months). Three patients developed distant metastases, including one with a late LR 13 years after resection. One disease-related death was recorded at the last follow-up. The 5-year and 10-year overall and DSS rates were both 83%. No separate PFS or DFS data were reported specifically for the NAC subgroup [26]. NAC regimens in MCS are often intensive. Young adult patients (who make up the majority of MCS) generally tolerate Ewing-like regimens, albeit with expected toxicities: myelosuppression (neutropenia in >50%, risk of febrile neutropenia), anaemia, thrombocytopenia, and typical chemo-specific effects (e.g., IFO-related encephalopathy or renal dysfunction, VCR neuropathy, anthracycline cardiotoxicity) [3,16,27,28]. In published series, treatment-related deaths are rare, but dose reductions/delays are sometimes required [13,29,30]. Close monitoring and growth factor support are standard. Where toxicity is a concern (older adults or patients with comorbidities), some teams use modified regimens (e.g., omitting ETO or using two-drug combinations such as DOX/IFO) to balance efficacy and safety. Common protocols include alternating cycles of VDC/IE (VCR, DOX, CTX/IFO, ETO) or similar combinations [13,29,31]. For example, a consensus approach is to alternate VDC (CTX, DOX VCR) with IE (IFO + ETO), mirroring the Ewing treatments. Some centres also use OSa-like regimens, e.g., DOX + CDDP or DOX + IFO in adults who cannot tolerate intensive paediatric regimens [29,31]. Overall, the risk–benefit balance favours NAC in fit adult patients.

## 4. Adjuvant Chemotherapy

In MCS, adjuvant systemic therapy is usually recommended after resection, in line with ES protocols [1,3]. Retrospective studies suggest that AC may improve both DFS and OS in MCS. For example, Cesari et al. compared AC with no CHT in 26 MCS patients [32]. Among patients who achieved complete surgical remission, the 5-year and 10-year continuous DFS was 76% in the CHT group versus 17% in the non-CHT group (*p* = 0.008). Overall, the 10-year OS rate was 21%. When all patients were considered, those who received CHT had a 10-year OS of 31% compared to 19% in those who did not receive CHT, although this difference was not statistically significant. The 10-year OS was 0% for patients metastatic at presentation versus 31% for localised disease; however, the study did not report earlier landmark OS (e.g., 2- or 5-year) by stage, limiting interpretability in the metastatic setting. Furthermore, patients with bone lesions had a 10-year OS of 29%, while none of the patients with soft tissue tumours survived to 10 years (*p* = 0.057). Separate data on PFS were not reported [32]. Notably, in a systematic review of 107 patients with MCS, the 5-year OS rate was 55%, and the 5-year event-free survival (EFS) rate was 45% [17]. AC, mainly based on anthracyclines, was evaluated in this series. Its use did not lead to a statistically significant improvement in OS (*p* = 0.139), whereas it was associated with a significant benefit in EFS (*p* = 0.046). The long-term outcomes were also reported, with OS rates of 43.5% at 10 years and 15.7% at 20 years, and EFS rates of 27.2% at 10 years and 8.1% at 20 years. These data suggest that while AC may delay disease progression, it does not significantly extend OS [17].

Although direct comparisons are retrospective (there are no randomised trials because of the rarity of the disease), the consistency of the results across studies has led to a consensus: AC is beneficial in MCS. Indeed, current expert guidelines explicitly state that MCS should be treated with surgery and CHT (plus RT in selected cases) as standard treatment [11,19,27]. Without CHT, MCS has a tendency to develop early distant metastases (especially to the lungs and other bones) in the first 2–3 years after surgery [13]. AC has been shown to significantly delay or reduce these metastases. Frezza et al. found that systemic CHT was associated with far fewer metastatic relapses in localised MCS [17]. In EMSOS, median PFS was 7.0 years across the localised-disease cohort; attribution of this figure specifically to adjuvant therapy is not supported, as the analysis was retrospective and combined peri-operative uses. By contrast, historical surgery-alone series suggest poorer long-term outcomes than contemporary multimodal cohorts; however, median PFS figures are often not reported separately. Five-year OS frequently does not exceed ~40–50% in these older series [2,15].

Given the typically young demographics, most MCS patients complete planned AC [31]. Toxicity profiles are similar to those described for NAC (myelosuppression is universal; cardiotoxicity, neuropathy, etc., are monitored). In the Frezza et al. study [17], treatment-related toxicity data were not detailed, but the implied tolerability was good as many received long courses. In smaller series, discontinuation of AC is usually due to either early relapse (despite therapy) or complications such as prolonged neutropenia/infection [14,17,28]. One challenge is that AC for bone sarcomas typically lasts several months—requiring patient motivation and support. Fortunately, as MCS often affects adolescents/young adults, aggressive therapy is usually feasible with modern supportive care.

Adjuvant regimens mirror the NAC approach (often the patient simply continues the ES-like regimen postoperatively) [33]. A common strategy is to alternate VCR-DOX-CTX and IFO-ETO for a total of ~6–12 months of therapy (including preoperative cycles). In some cases where NAC has not been given (e.g., unplanned excision or unexpected diagnosis after surgery), AC is started as soon as wound healing allows [12,13,14,30,33,34]. Anthracycline-based combinations are considered essential; single-agent treatments are considered inadequate given the aggressive nature of the tumour [33].

## 5. First-Line Systemic Palliative Therapy

For metastatic or unresectable MCS in adults, multi-agent CHT is an important treatment option. Given the known chemosensitivity of MCS, multi-agent regimens similar to those used for localised disease may be used. In published series, most MCS patients received combination CHT [12,29,35]. In a retrospective multicentre study evaluating palliative first-line systemic therapy for unresectable CS, 25 patients (22% of the cohort) were diagnosed with MCS and uniformly received combination CHT regimens [29]. The overall median PFS in these MCS patients was 6.7 months. Notably, among the chemotherapeutic strategies, patients treated with an IFO-DOX regimen (IFO + DOX; n = 4) achieved a mean PFS of 3.7 months, while those treated with a cisplatin-DOX regimen (CDDP + DOX; n = 4) demonstrated a significantly superior mean PFS of 7.7 months (*p* = 0.04) [29]. Similarly, Italiano et al. (2013) reported that of advanced MCS cases treated in France, most received polychemotherapy (often based on IFO/DOX or CTX), with an objective response rate of 31% [12]. The overall treatment strategy was cytotoxic regimens, with 73% of the cohort receiving anthracycline-based CHT and 54.5% receiving combination therapy. Notably, the ORR for MCS was 31%, a figure significantly superior to that observed for dedifferentiated (20.5%), conventional (11.5%) and clear cell CS (0%) (*p* = 0.04). Although the median PFS for the entire study population was 4.7 months [95% CI: 3–6.4] and the median OS was 18 months [95% CI: 14.5–21.6], these overall figures highlight a relative chemosensitivity in MCS. Furthermore, previous studies in paediatric and young adult cohorts (e.g., the CWS and COSS groups) have reported ORRs as high as 57% in the neoadjuvant setting, suggesting that the intrinsic biology of MCS may confer a higher responsiveness to cytotoxic agents [12]. Moreover, in a reported case of retroperitoneal MCS with iliac vein metastases, a 50-year-old woman underwent first-line palliative CHT with a combination regimen of DOX and IFO [36]. The patient received five cycles of DOX + IFO—an anthracycline-based regimen that serves as the cytotoxic backbone in the palliative treatment of high-grade soft tissue sarcomas. Although specific dosing parameters were not detailed, this regimen is consistent with standard protocols used in the treatment of sarcoma. Surgical resection was recommended following these cycles, but was not performed due to poor compliance. Disease progression then occurred, leading to the administration of further lines of therapy (including a multi-agent regimen of VCR, CTX, and DOX, and later single-agent GEM. Nevertheless, the initial palliative approach with DOX + IFO highlights its role in the treatment of advanced MCS, despite the overall aggressive disease course and limited durability of response [36].

## 6. Second- and Further Lines of Palliative Systemic Treatment

If MCS progresses after first-line CHT, available treatment options include second-line cytotoxic regimens or the use of targeted agents, although data are sparse and there is no consensus for this setting. Given the rarity of MCS, there is no standard second-line treatment, but clinicians extrapolate from other sarcomas [12,37,38]. Common second-line choices include IFO (if not used in the first-line setting) either alone or with ETO, GEM + docetaxel, and even high-dose methotrexate in selected cases (especially if initial therapy was not MTX). Italiano et al. noted that responses were seen “in the second-line setting in patients with primary resistance to anthracyclines”, suggesting that agents such as IFO or GEM may have activity after DOX failure [12]. In fact, they observed some objective responses to second-line IFO or GEM-based therapy, suggesting that these drugs have modest efficacy in chemo-refractory CS. Moreover, a case report describes a 39-year-old man with extraskeletal MCS that had metastasised to the pancreas, bone, and lung [38]. The patient underwent surgical resection of the primary tumour and metastases. He then received AC, which initially improved his condition. This first CHT regimen included three cycles of IFO and DOX, three cycles of cisplatin and DOX, and two cycles of VDC. At the end of this treatment, most of the metastatic lesions were no longer detectable by positron emission tomography. Fifteen months after surgery, the metastatic tumours relapsed. This interval reflects a PFS of about 15 months after the initial treatment. After relapse, the patient received additional (second and subsequent) palliative CHT. This later regimen consisted of one cycle of IFO and DOX, one cycle of IE, one cycle of VDC, and finally four cycles of GEM and docetaxel. The additional palliative CHT did not control tumour growth. The patient’s OS from the time of surgery was 34 months. The authors do not provide a separate measure of DFS after second-line palliative treatment, but initial DFS can be inferred from the 15-month PFS period [38]. In another case report after several relapses and widespread metastases, the patient was treated with palliative CHT using a MAID regimen for six cycles [39]. The regimen included mesna, adriamycin, IFO, and dacarbazine. After palliative CHT, the follow-up CT scan showed SD. The patient continued to receive systemic CHT for further disease control [39]. Araki et al. describe trabectedin as a second- or later-line palliative treatment in patients with advanced translocation-related sarcomas, which included MCS [40]. In the crossover group (patients who initially received best supportive care), 29 patients received trabectedin at a dose of 1.2 mg/m^2^ every 3 weeks. The median PFS in these patients was 7.3 months (95% CI: 2.9–9.1 months), compared with 0.9 months (95% CI: 0.9–1.0 months) in the best supportive care arm. The median OS after crossover was 10.3 months (95% CI: 6.6—not reached) at the cut-off date. The growth modulation index (GMI), defined as the ratio of time to progression on trabectedin to that on previous treatment, was ≥1.33 in 86% of patients, suggesting a meaningful benefit from trabectedin. ORR was 14% and the overall disease control rate (DCR, including CR, PR, and SD) was 69% across all translocation-related sarcomas. In TRS studies that included very small EMCS/MCS subsets, crossover to trabectedin after best supportive care improved PFS in the overall TRS cohort; EMCS/MCS numbers were <10 in sub-analyses, precluding firm MCS-specific conclusions. Therefore, the efficacy outcomes for MCS are incorporated into the overall results of the trial. No separate data for DFS were provided [40]. One of the responses to second-line CHT was noted recently in extraskeletal MCS of the elbow [41]. After progression on first-line CHT, GEM-DTX achieved PR (RECIST 1.1); later progression was treated with VAI × 7 with a second PR. Isolated mediastinal PD was refractory to RT 45 Gy/15. Rechallenge with TMZ-IRI and GEM-DTX was ineffective. Subsequent disseminated progression included liver and central nervous system (CNS) M1, and death occurred approximately four years after diagnosis, following eight lines of systemic therapy. This trajectory illustrates transient responses in ≥2L-M and supports trial enrolment plus selective local control for oligoprogression [41].

## 7. Molecular Targets and Therapies Under Investigation

Emerging targeted therapies show additional promise. For Xie et al. [42], in a two-centre retrospective series of 33 CS patients, including 4 with MCS, apatinib achieved an overall 3-month DCR of 78.79% and overall median PFS of 12.4 months; subtype-specific DCR or PFS for MCS were not reported. Among the four MCS cases, ORR was 25% (1/4). Ivosidenib—IDH1 inhibitor, commonly mentioned regarding novel CS therapies—showed disease control in IDH1-mutant conventional chondrosarcoma in Phase I (DCR 73.5%, median PFS 5.6 months), but MCS seldom harbours IDH1 mutations and may require alternative therapeutic approaches. Ivosidenib’s role in MCS is presently unclear [43]. The significant toxicity associated with CHT regimens poses a major challenge in MCS treatment. Anthracycline-based therapies are known for their cardiotoxic effects, particularly at cumulative doses exceeding 450 mg/m^2^ of DOX. In contrast, targeted agents like apatinib and pazopanib offer more favorable toxicity profiles. In terms of immunotherapies, which are commonly associated with reduced toxicity, the knowledge is limited. One retrospective study [44] analysed clinical data from 28 patients with metastatic or locally advanced soft tissue or bone sarcoma who were treated with nivolumab, either alone or in combination with pazopanib. Of these patients, 50% experienced clinical benefit, defined as a partial response or SD, after at least four cycles of nivolumab. These results support the need for further investigation into the efficacy of nivolumab and other checkpoint inhibitors as potential treatments for sarcoma. Notably, a metastatic MCS case achieved a sustained partial response for ~12 months on the multi-tyrosine kinase inhibitors cabozantinib, supporting VEGFR/MET inhibition as a tractable option [45]. Also, the molecular characteristics of MCS have a significant impact on treatment outcomes. The HEY1-NCOA2 fusion gene, a hallmark of MCS, drives tumour growth through PI3K/AKT and BCL2 pathways, contributing to resistance to standard therapies. Agents targeting these pathways, such as BCL2 inhibitors and PI3K/mTOR inhibitors, are under investigation and may offer new opportunities to improve the efficacy of CHT. Studies by Huvos et al. [21] and Italiano et al. [12] suggest that well-differentiated tumours and lower tumour burden at diagnosis are associated with better OS and DFS. Conversely, high Ki67 indices and advanced stage disease predict poorer outcomes. The novel study [46] investigated the role of KDELR1, a receptor involved in protein trafficking between the endoplasmic reticulum and the Golgi, in regulating drug resistance and malignant behaviour in CS. The researchers analysed CS samples using single-cell RNA sequencing to create a transcriptional map and compared it with OSa and normal bone tissue. They found that KDELR1 was highly expressed in CS cells, particularly in high-grade tumours, and that its levels correlated with poor prognosis, higher LR rates and greater metastatic potential. KDELR1 was shown to affect cell survival, proliferation, and migration by modulating the Hippo-YAP1 signalling pathway. The Hippo signalling pathway is important in regulating cell growth and its effector, YAP1, promotes cancer progression when active in the nucleus. High KDELR1 expression led to reduced phosphorylation of YAP1, allowing it to remain active and drive tumour growth. In contrast, reducing KDELR1 levels increased YAP1 phosphorylation, causing YAP1 to accumulate in the cytoplasm in an inactive state, thereby suppressing cancer cell growth and improving drug sensitivity. The study also found that KDELR1 interacts with integrins—cell surface proteins involved in cell adhesion and signalling—through the ITG-PLCγ-MAP4K4 axis. This interaction regulates the Hippo signalling pathway, which links extracellular signals to intracellular growth mechanisms. Knockdown of KDELR1 disrupted this axis, reducing integrin activity and promoting YAP1 inactivation. Animal studies confirmed these findings. Tumours with reduced KDELR1 expression showed slower growth, reduced metastatic activity, and improved response to the CHT drug cisplatin. Introducing an active YAP1 mutant reversed these effects, further demonstrating the importance of KDELR1 in maintaining YAP1 activity and cancer aggressiveness. Targeting KDELR1 may enhance the efficacy of CHT and inhibit tumour progression by restoring the tumour suppressor activity of the Hippo-YAP1 pathway.

## 8. Conclusions

MCS is characterised by small round cell biology, early haematogenous spread and relative chemosensitivity (Table 3). Across contemporary series, 5-year OS typically spans ~55–73%, with the most consistent systemic-therapy signal being improved EFS/DFS rather than OS. In localised disease, peri-operative multi-agent Ewing-type CHT integrated with complete resection is associated with reduced relapse risk and longer disease control, e.g., EMSOS: HR for recurrence 0.482 (95% CI 0.213–0.996); HR for death 0.445 (95% CI 0.256–0.774) [12]. Radiographic tumour shrinkage with NAC is uncommon and stable disease predominates; nevertheless, NAC may confer advantages of early micrometastatic control and facilitate margin-negative resection in selected cases [11]. AC is expert-consensus recommended and guideline-endorsed; pooled and multi-institutional data suggest delayed relapse and longer EFS, while OS gains remain less certain given ultra-rare disease constraints and retrospective designs. In advanced MCS, first-line polychemotherapy produces modest, non-curative activity (ORR ~25–35% in adults; median PFS ~4.7–6.7 months), with higher activity reported in paediatric/AYA subsets. Due to the significant haematological, cardiac, renal and neurotoxic risks associated with Ewing-type regimens, careful patient selection, individualised dosing and multidisciplinary discussions are essential. Priority research directions include prospective, MCS-specific cohorts with predefined response and necrosis criteria; adaptive peri-operative strategies aligned to early response; and biomarker-guided trials of anti-angiogenic TKIs, trabectedin within translocation-related frameworks, and BCL2 or PI3K–mTOR inhibition. The establishment of international registries is crucial for generating precise estimates of effect and refining patient selection.

## Figures and Tables

**Table 2:** NCCN and ESMO Guidelines for the treatment of mesenchymal chondrosarcoma.

Clinical Context	Modality	NCCN [20]	ESMO [19]
Localised, resectable	Surgery	Wide excision with negative margins in reference centres/MDTs	Wide excision with negative margins in reference centres/MDTs
	Systemic therapy	Treat as Ewing sarcoma; apply Ewing-type multi-agent peri-operative chemotherapy. NAC used selectively for downstaging/early micrometastatic control; AC reasonable to complete peri-operative intent	Consider peri-operative multi-agent chemotherapy given relative chemosensitivity; NAC selectively to assist resection; AC reasonable based on risk, response and tolerance
	Radiotherapy	Consider RT for unresectable disease or close/positive margins; particle therapy where available for challenging sites	Consider RT for unresectable or anatomically challenging sites; particle therapy where available
Unresectable or metastatic	Systemic therapy	Polychemotherapy for disease control per Ewing practice; prioritise clinical-trial/registry enrolment	Polychemotherapy for disease control; prioritise trial/registry enrolment given ultra-rarity
	Local control	Surgery or RT for symptom relief and selected oligometastatic control where feasible	Surgery or RT for symptom relief and selected oligometastatic control where feasible
Follow-up	Surveillance	Structured surveillance per bone-sarcoma pathways; monitor late effects of surgery/RT/systemic therapy	Structured surveillance per bone-sarcoma pathways; monitor late effects of surgery/RT/systemic therapy

The guidelines align management with Ewing sarcoma pathways. For localised, resectable disease, negative-margin surgery is central, and peri-operative multi-agent chemotherapy is recommended using Ewing-type protocols. NAC is used selectively for downstaging, and AC is used to achieve the intended outcome. RT is considered for unresectable or marginally resectable sites. For unresectable or metastatic disease, polychemotherapy is palliative, and local control is employed to manage symptoms or oligometastatic sites. Structured surveillance is advised. AC—adjuvant chemotherapy; ESMO—European Society for Medical Oncology; MDT—multidisciplinary team; NAC—neoadjuvant chemotherapy; NCCN—National Comprehensive Cancer Network; RT—radiotherapy.

**Table 3:** Chemotherapy regimens used in mesenchymal chondrosarcoma.

Regimen	Chemotherapeutics with Dosing	Setting(s)	Frequency Given	References
VDC	VCR 1.4–2.0 mg/m^2^ d1 (cap 2 mg) + DOX 60–75 mg/m^2^ d1 ± split + CTX 1.2 g/m^2^ d1	NA, ADJ, 1L-M	q2-3w	[29]
IE	IFO 1.8–2.0 g/m^2^ d1–5 + ETO 100 mg/m^2^ d1–5	NA, ADJ, 1L-M	q3w (often q2w in EwS)	[16,29]
VDC/IE	As above, alternating VDC with IE	NA, ADJ, 1L-M	q2-3w	[13,47]
VIDE	VCR 1.4–1.5 mg/m^2^ d1 + IFO 3 g/m^2^ d1–3 + DOX 20 mg/m^2^ d1–2 + ETO 150 mg/m^2^ d1–3	NA, 1L-M	q3w	[29,48]
VAC	VCR 1.5 mg/m^2^ d1 + ACT-D 0.5–1.25 mg/m^2^ d1 ± d3,5 + CPA 1.2–2.0 g/m^2^ d1	ADJ, 1L-M	q3w	[29]
VAI	VCR 1.5 mg/m^2^ d1 + ACT-D 0.5–0.75 mg/m^2^ d1–2 + IFO 3 g/m^2^ d1–2	ADJ, 1L-M	q3w	[29]
AI	DOX 20–25 mg/m^2^ d1–3 + IFO 2.5–3 g/m^2^ d1–3	1L-M	q3w	[12]
AP	DOX 60–75 mg/m^2^ d1 + CDDP 80–100 mg/m^2^ d1–3	ADJ, 1L-M	q3w	[12]
API	DOX 60 mg/m^2^ d1 + IFO 5 g/m^2^ d1 + CDDP 100 mg/m^2^ d2	1L-M	q3w	[12]
IP	IFO 2.5–3 g/m^2^ d1–3 + CDDP 80–100 mg/m^2^ d1	1L-M	q3w	[12]
DOX (mono)	DOX 50–75 mg/m^2^ d1	1L-M	q3w	[12]
PLD (mono)	PLD 40–50 mg/m^2^ d1	≥2L-M	q4w	[12]
IFO (mono)	IFO 5–9 g/m^2^ d1 or divided	≥2L-M	q3w	[12]
GEM (mono)	GEM 1000 mg/m^2^ d1,8,15	≥2L-M	q4w	[12,49]
GEM-DTX	GEM 900–1000 mg/m^2^ d1,8 + DTX 75–100 mg/m^2^ d8	≥2L-M	q3w	[12]
DTIC (mono)	DTIC 850–1000 mg/m^2^ d1 or 200–250 mg/m^2^ d1–5	≥2L-M	q3-4w	[13]
ETO (oral)	ETO 40–50 mg/m^2^ PO d1–21	≥2L-M	q4w	[12]
TMZ-IRI ± VCR (VIT)	TMZ 100 mg/m^2^ d1–5 + IRI 50 mg/m^2^ d1–5 ± VCR 1.5 mg/m^2^ d1	≥2L-M	q3w	[12]
TMZ (mono)	TMZ 150–200 mg/m^2^ d1–5	≥2L-M	q4w	[50]
TRB	TRB 1.2–1.5 mg/m^2^ 24-h infusion d1	≥2L-M	q3w	[37,51,52]
VIP	ETO 100 mg/m^2^ d1–3 + IFO 1.5 g/m^2^ d1–5 + CDDP 60 mg/m^2^ d1	1L-M	q3w	[13,53]
ICE	IFO 1.8 g/m^2^ d1–5 + CBDCA AUC 5–6 d1 + ETO 100 mg/m^2^ d1–5	≥2L-M	q3w	[29]
MAP	MTX high-dose 8–12 g/m^2^ d1 + DOX 75 mg/m^2^ + CDDP 120 mg/m^2^	ADJ, NA	q3-4w	[14,29]
MAID	Mesna-DOX-IFO-DTIC, typically 72-h CI: DOX 20 mg/m^2^/d, IFO 2000 mg/m^2^/d, DTIC 250 mg/m^2^/d	1L-M, ≥2L-M	q3w	[29]
CYVADIC	CTX 500 mg/m^2^ d1 + VCR 1.5 mg/m^2^ d1 + DOX 50 mg/m^2^ d1 + DTIC 400 mg/m^2^ d1–3	1L-M, ≥2L-M	q4w	[29]
TC	TOP (topotecan) 0.75 mg/m^2^ d1–5 + CTX 250 mg/m^2^ d1–5	≥2L-M	q3w	[29]

Catalogue of first-line peri-operative and metastatic backbones, plus later-line options, with representative doses and schedules used in practice. Ewing-type combinations predominate peri-operatively (VDC/IE; VIDE), while metastatic therapy ranges from anthracycline–alkylator pairs (AI, AP) to gemcitabine–docetaxel and temozolomide-based regimens in ≥2L-M settings. Frequencies are presented as given (q2-4 weeks) and should be individualised for performance status, organ function, and prior dose intensity. Abbreviations: 1L-M—first-line metastatic; ≥2L-M—second-line or later metastatic; ADJ—adjuvant; AI—doxorubicinifosfamide; AP—doxorubicin-cisplatin; API—doxorubicin-ifosfamide-cisplatin; AUC—area under the curve; CBDCA—carboplatin; CDDP—cisplatin; CI—continuous infusion; CTX/CPA—cyclophosphamide; DOX—doxorubicin; DTIC—dacarbazine; DTX—docetaxel; ETO—etoposide; GEM—gemcitabine; ICE—ifosfamidecarboplatin-etoposide; IE—ifosfamide-etoposide; IFO—ifosfamide; IP—ifosfamide-cisplatin; IRI—irinotecan; MAP—high-dose methotrexate-doxorubicin-cisplatin; MAID—mesna-doxorubicin-ifosfamide-dacarbazine; MTX—methotrexate; NA—neoadjuvant; PLD—pegylated liposomal doxorubicin; q2w/q3w/q4w—every 2/3/4 weeks; TC—topotecan-cyclophosphamide; TMZ—temozolomide; TMZ-IRI—temozolomide-irinotecan; TOP—topotecan; TRB—trabectedin; VAC—vincristine-actinomycin D-cyclophosphamide; VAI—vincristinedactinomycin-ifosfamide; VCR—vincristine; VDC—vincristine-doxorubicin-cyclophosphamide; VDC/IE—vincristine-doxorubicin-cyclophosphamide alternating with ifosfamide-etoposide; VIDE—vincristine-ifosfamidedoxorubicin-etoposide; VIP—etoposide-ifosfamide-cisplatin; VIT—temozolomide-irinotecan-vincristine.

## Data Availability

No new data were created or analyzed in this study. Data sharing is not applicable to this article.
